# *In Vitro* Behavior of Human Adipose Tissue-Derived Stem Cells on Poly(*ε*-caprolactone) Film for Bone Tissue Engineering Applications

**DOI:** 10.1155/2015/323571

**Published:** 2015-10-08

**Authors:** Cecilia Romagnoli, Roberto Zonefrati, Gianna Galli, Dario Puppi, Alessandro Pirosa, Federica Chiellini, Francesco Saverio Martelli, Annalisa Tanini, Maria Luisa Brandi

**Affiliations:** ^1^Department of Surgery and Translational Medicine, University of Florence, 50139 Florence, Italy; ^2^BIOLab Research Group, Department of Chemistry and Industrial Chemistry, University of Pisa, UdR INSTM Pisa, 56124 Pisa, Italy; ^3^Microdentistry Institute, 50123 Florence, Italy

## Abstract

Bone tissue engineering is an emerging field, representing one of the most exciting challenges for scientists and clinicians. The possibility of combining mesenchymal stem cells and scaffolds to create engineered tissues has brought attention to a large variety of biomaterials in combination with osteoprogenitor cells able to promote and regenerate bone tissue. Human adipose tissue is officially recognized as an easily accessible source of mesenchymal stem cells (AMSCs), a significant factor for use in tissue regenerative medicine. In this study, we analyze the behavior of a clonal finite cell line derived from human adipose tissue seeded on poly(*ε*-caprolactone) (PCL) film, prepared by solvent casting. PCL polymer is chosen for its good biocompatibility, biodegradability, and mechanical properties. We observe that AMSCs are able to adhere to the biomaterial and remain viable for the entire experimental period. Moreover, we show that the proliferation process and osteogenic activity of AMSCs are maintained on the biofilm, demonstrating that the selected biomaterial ensures cell colonization and the development of an extracellular mineralized matrix. The results of this study highlight that AMSCs and PCL film can be used as a suitable model to support regeneration of new bone for future tissue engineering strategies.

## 1. Introduction

The rapidly growing knowledge of cell biology and material science has brought attention to the tissue engineering approach as a promising strategy, alternative to the conventional autogenic or allogenic surgical techniques for bone defects regeneration [1–3]. Tissue engineering is a multidisciplinary field that is affected by three main agents including cell source, scaffold biomaterial, and biochemical agents. In particular, bone tissue engineering involves the transplant of an appropriate scaffolding biomaterial alone or in combination with cells, that is, osteoblast progenitors and/or growth factors capable of osteogenic differentiation necessary for bone repair and regeneration [3, 4].

In order to achieve the proposed aim, it is important to select appropriate cell sources and biomaterials, since the cell-scaffold interaction represents a crucial step for the success of tissue engineering applications. One of the efforts in this field is the use of mesenchymal stem cells (MSCs) as a source for tissue engineering [5]. MSCs are undifferentiated, nonhematopoietic cells of mesodermal derivation that are present in a number of postnatal organs and connective tissues, including bone marrow, trabecular bone, synovium, umbilical cord, dental tissues, and skin [6–10], and have the potential to differentiate into osteogenic, chondrogenic, adipogenic, and endothelial lineages [11–14]. Bone marrow mesenchymal stem cells (BMSCs) represent the most thoroughly studied source of MSCs for bone tissue regeneration. However, bone marrow is obtained through an invasive and painful procedure.

In recent years, a promising population of MSCs has been identified within adipose tissue, termed adipose-derived mesenchymal stem cells (AMSCs). In fact, human adipose is ubiquitous and can easily be obtained in large quantities with very little donor site morbidity or patient discomfort. Moreover, stem cell yields are greater from adipose tissue than from other stem cell reservoirs, a significant factor for use in regenerative medicine, as many 1 × 10^7^ AMSCs can routinely be isolated from 300 mL of lipoaspirate, with greater than 95% purity [15–18].

AMSCs have been extensively described in terms of stemness [19, 20] and osteogenic differentiation capacity [18, 21], and many researchers have shown the potential use of AMSCs in combination with a variety of different materials, including ceramics, titan alloys, natural and synthetic polymers, and natural or semisynthetic grafts for bone tissue engineering [21–27], proposed for the treatment of bone pathological conditions.

In our study, we decided to utilize the polyester poly(*ε*-caprolactone) (PCL) in the form of film prepared by solvent casting, since it is a nontoxic, biodegradable polymer, with a slow degradation rate (months to years), which demonstrates good mechanical strength and biocompatibility [28–32]. PCL has been widely investigated for different biomedical applications, such as drug delivery systems (nano- or microparticles), medical devices (e.g., sutures, wound dressing, contraceptive devices, and internal fixation devices), and tissue engineering [33, 34].

Despite the publication of numerous reports on PCL for bone tissue engineering destined for biomedical applications [35–40], there is no literature describing the* in vitro* behavior in terms of adhesion, vitality, proliferation, and osteogenic differentiation of human AMSCs (hAMSCs) seeded onto PCL film. Therefore, the objective of the study was to demonstrate the potential of this cell-biomaterial model for the proposed application, supporting and promoting the research on hAMSCs and PCL in bone tissue engineering strategies.

## 2. Materials and Methods

### 2.1. hAMSCs Cultures

hAMSCs line, named PA42, was isolated from small fragments of subcutaneous adipose tissue biopsy obtained during general surgery from a patient, after signing an informed consent in accordance with a protocol approved by the Local Ethics Committee of AOU-Careggi, Firenze (Italy), for human studies (Rif.n.31-13). Briefly the adipose tissue sample was minced into small pieces (0.5–1 mm) and digested for 3 hours at 37°C in Ham's F12 Coon's modification medium supplemented with 20% fetal bovine serum (FBS) and 3 mg/mL collagenase type I (C-0130, Sigma Aldrich). The tissue was then mechanically dispersed by pipetting and passed through a sterile 230 *μ*m stainless steel tissue sieve. The undigested tissue trapped in the sieve was discarded, while the infranatant containing the hAMSCs fraction was collected and the cells were sedimented by centrifugation at 300 g for 5 min. The cells were resuspended and primary culture was seeded in 100 mm tissue culture plates at 37°C in humidified atmosphere with 5% CO_2_ in medium thus composed: Ham's F12 Coon's modification medium supplemented with 20% FBS, 100 IU/mL penicillin, 100 *μ*g/mL streptomycin, and 1 ng/mL basic Fibroblast Growth Factor (bFGF). The medium was refreshed twice a week and the cells were used for further subculturing or cryopreservation upon reaching 5 × 10^3^ cells/cm^2^. From this point, primary culture was expanded in growth medium (GM), Ham's F12 Coon's modification medium supplemented with 10% FBS, 100 IU/mL penicillin, 100 *μ*g/mL streptomycin, and 1 ng/mL basic Fibroblast Growth Factor (bFGF), and/or differentiated in specified medium according to the experimental protocol.

### 2.2. hAMSCs Cloning

PA42 cells at the 3rd passage were used for cell cloning. Cells in an active phase of growth were cloned by the dilution plating technique. Cells were detached with trypsin 1 : 250 0.4 mg/mL in Dulbecco's Phosphate Buffered Saline (DPBS) without Ca^2+^ without Mg^2+^ with EDTA 0.2 mg/mL and with glucose 1 mg/mL, resuspended in Coon's medium + 20% FCS. The cell suspension was diluted to a concentration of 10 cells/mL in the following cloning medium: Coon's and 20% FCS supplemented with 25% conditioned medium prepared from human fetal fibroblast culture. The cell suspension was maintained in agitation and 0.1 mL was rapidly distributed per well of a 96-well, half area tissue culture plate. Each well was carefully observed and the wells containing only one cell were scored. The cloning culture was incubated at 37°C in humidified air with 5% CO_2_. When colonies reached the consistency of 500–1000 cells, they were detached, collected, and first transferred in 24-well plates and subsequently expanded in 60 mm and 100 mm dishes.

### 2.3. Soft Agar Assay for Neoplastic Transformation

Neoplastic transformed cells form colonies that grow progressively in soft agar. A 35 mm dish was coated with 1% agar prepared in culture medium maintained liquid at 45°C. The dish was immediately cooled. Cells in growth phase were detached, suspended in medium, diluted to double the required final concentration, and maintained at 37°C. 0.68% agar was prepared in medium and maintained at 45°C. Cell suspension was mixed with an equal volume of 0.68% agar, distributed into the agar coated dish to obtain a final concentration of 2.5 × 10^3^ cells/dish, and immediately cooled. The cells were cultured at 37°C in humidified air with 5% CO_2_ for 3-4 weeks until the formation of colonies and their growth. Colonies formed per dish were observed and counted in phase contrast microscopy.

### 2.4. hAMSCs Characterization and Multipotency Evaluation

The characterization of the PA42 cell line and the finite clonal cell line, named PA42-C4, was performed by immunocytochemical staining of the main stemness markers of mesenchymal stem cells (CD44, CD105, and STRO1) and by studying their multipotency toward both the adipogenic and osteogenic phenotypes, as described below.

#### 2.4.1. Immunocytochemical Staining

PA42-C4 finite clonal line was seeded in 24-well plate and grown in GM until 23–30% confluence. Then, cells were washed with DPBS (two times), fixed in 4% paraformaldehyde PFA/DPBS for 10 min, and washed with ultrapure water (three times). After permeabilization with 0.2% PBS 1x/Triton X-100 solution for 15 min and treatment with RNAse, samples were incubated with primary antibody for CD44, CD105, STRO1, and CD45 (all Invitrogen) in PBS at 4°C, overnight. The following day, primary antibody was removed by extensive washes with PBS and secondary antibody Alexa Fluor 488-conjugated (Invitrogen) was incubated for 1 h at room temperature in the dark. Subsequently, samples were washed with PBS and observed by Laser Scanning Confocal Microscopy (LSCM).

#### 2.4.2. Adipogenic Differentiation

PA42 cell line and PA42-C4 finite clonal line were cultured with a specific adipogenic medium (AM): Ham's F12 Coon's modification medium supplemented with 10% (FBS), 100 IU/mL penicillin, 100 *μ*g/mL streptomycin and 1 *μ*M dexamethasone, 1 *μ*M bovine insulin, and 0.5 mM isobutylmethylxanthine (IBMX). The medium was refreshed twice a week. The expression of the adipogenic phenotype was evaluated on cells cultured in AM or GM for 20 days by Oil Red O staining.

#### 2.4.3. Osteogenic Differentiation

PA42 cell line and PA42-C4 finite clonal line were plated on tissue culture dishes at a cell density of 1 × 10^4^ cells/cm^2^ in GM and grown to 70–80% confluence. Afterwards, the medium was switched to osteogenic medium (OM): Ham's F12 Coon's modification medium supplemented with 10% FBS, 100 IU/mL penicillin, 100 *μ*g/mL streptomycin, 10 nM dexamethasone, 0.2 mM sodium L-ascorbyl-2-phosphate, and 10 mM *β*-glycerol phosphate. The medium was refreshed twice a week. The expression of the osteoblastic phenotype was evaluated at 10 and 20 days from induction by contemporary monitoring ALP activity and mineralization by cytochemical staining. For ALP staining, the cells were washed with DPBS (two times), stained with a specific dye mixture: 5 mg Naphthol-AS-MX phosphate sodium salt, previously dissolved in 1 mL dimethyl sulfoxide, 40 mg Fast Red Violet LB, or Fast Blue BB, dissolved in 49 mL Tris-HCl Buffer 280 mM, pH 9.0 for 30 min at 37°C. Then, the cells were washed with DPBS (two times), fixed in 4% paraformaldehyde (PFA)/DPBS for 15 min, and washed with ultrapure water (three times). ALP+ cells were stained in red and nuclei counterstained in blue with Mayer's acid hemalum or with DAPI. For mineralization staining, the cells were washed with DPBS (two times), fixed in 4% PFA/DPBS for 15 min, and washed with ultrapure water (three times). Calcium mineral deposits were stained for 2 min with 2% Alizarin Red S, pH 6.0, and rinsed with water; calcium mineralized deposits were stained in red-orange.

### 2.5. Preparation of PCL Film

Poly(*ε*-caprolactone) (PCL, CAPA 6800, MW = 80000 g/mol) was kindly supplied by Perstorp Caprolactones Ltd. (UK).

PCL solution at a concentration of 5% w/v was prepared by dissolving the polymer in acetone (Sigma Aldrich) at 30°C under gentle stirring overnight. PCL film was prepared by solvent casting of the polymeric solution. For this purpose 8 mL of the solution was placed in a 12 cm diameter glass Petri dish. The casted solution was left overnight in an acetone closed chamber and dried for 1 day under a fume hood. The dried film was removed from the Petri dish and left under the fume hood for 24 h and kept under vacuum for 8 h to eliminate residual solvent. Afterwards, the PCL film was divided into small round disks with a diameter of 14 mm, sterilized overnight in 70% ethanol, and extensively washed in PBS.

Before cell seeding, all disks were incubated overnight in GM, in order to promote and facilitate cell adhesion.

### 2.6. Investigation of Adhesion of hAMSCs on PCL Film

Adhesion of hAMSCs, PA42-C4 finite clonal line, grown on the PCL film, was investigated by analysis of fibronectin, the main cell adhesion protein, and observation of cytoskeleton fibers, using LSCM. hAMSCs were seeded on PCL films, positioned in a 24-well plate, at a cell density of 5 × 10^3^ cells/well, and cultured in GM for 7 days. Afterwards, samples were washed with DPBS (two times), fixed in 4% paraformaldehyde (PFA/DPBS) for 10 min, and washed with ultrapure water (three times). After permeabilization with 0.2% PBS 1x/Triton X-100 solution for 15 min, samples were treated with RNAse in 2% bovine serum albumin (BSA) in order to degrade RNA and to block nonspecific sites.

Afterwards, samples were incubated with primary antibody for fibronectin (Sigma Aldrich) in PBS at 4°C, overnight. The following day, primary antibody was removed by extensive washes with PBS, and secondary antibody Alexa Fluor 488-conjugated (Invitrogen) was incubated for 1 h at room temperature in the dark. Cytoskeleton fibers (F-actin) were stained with phalloidin-Alexa Fluor 635 (Invitrogen) for 45 min a room temperature in the dark. Subsequently, samples were washed with PBS and observed by Laser Scanning Confocal Microscopy (LSCM).

### 2.7. Cell Viability and Proliferation on PCL Film

Viability of the PA42-C4 clonal line and cytocompatibility on biomaterial were assessed by staining of the samples with Acridine Orange (AO). Cells were seeded on PCL film in 24-well plate at a density of 2 × 10^4^ cells and vitality was assayed at specific end points (2, 4, 7, 10, 13, and 16 days). Samples were washed three times with DPBS to remove dead cells and serum proteins. Afterwards, they were incubated in 4 mg/L AO solution (in PBS, 2 mL/well) in the dark at room temperature for 10 min and washed three times with PBS. The cells were immediately observed in phase contrast and under fluorescence (BP365/FT395/LP397 filter set) with an Axiovert 200 M microscope, and images were acquired with Axiovision Software on an AxioCam HRC 12 megapixel camera (Carl Zeiss, Oberkochen, Germany). When stained with AO, DNA and mitochondria emit metachromatic green fluorescence (530 nm), and lysosomes, typically present in suffering or damaged cells, emit red fluorescence (650 nm) following excitation by ultraviolet (UV) light (365 nm).

The proliferation rate of the PA42-C4 clonal line on biomaterial was evaluated via 3-(4,5-dimethylthiazol-2-yl)-2,5-diphenyl-tetrazolium bromide (MTT; Sigma Aldrich, USA) assay. Cells were seeded on PCL film in a 24-well plate at a cell density of 5 × 10^3^ and incubated at 37°C and 5% CO_2_. After 2, 4, 7, 10, 13, and 16 days of cell culturing, samples were washed in DPBS and l mL of MTT solution was added to each well. For conversion of MTT to formazan crystals by mitochondrial dehydrogenases of living cells, the plate was incubated at 37°C for 4 h. For dissolution of the dark-violet intracellular formazan, the supernatant was removed and 300 *μ*L of 0.04 N HCl in isopropanol, as solvent, was added per well and plate shaken for 20 min. The optical density was read at a wavelength of 570 nm in cuvette using BioPhotometer (Eppendorf). The same procedure was performed for cells cultured on tissue culture polystyrene as control. Each experimental point was performed in triplicate.

### 2.8. ALP Assay and Mineralization of hAMSCs on PCL Film

The PA42-C4 clonal line was seeded on PCL film, positioned in a 24-well plate, at a concentration of 1 × 10^5^ cells/well. After a week, the GM was replaced with OM containing the fluorophore calcein 1 *μ*g/mL and incubated from 7 to 35 days. At the end of the incubation, the cells were washed with DPBS (two times), fixed in 4% PFA/DPBS for 15 min, washed with ultrapure water (three times), dried, and preserved at 4°C until analysis. Each experimental point was performed in quadruplicate and each experiment repeated three times.

#### 2.8.1. ALP Assay

Each well containing differentiated PA42-C4 cells on PCL film was incubated with 500 *μ*L of 4-methylumbelliferyl phosphate in 280 mM Tris-HCl Buffer, pH 9.0 for 30 min at 37°C. The reaction was stopped by the addition of 2.5 mL 0.1 M NaOH. ALP activity was measured with a spectrofluorometer LS55 (PerkinElmer) at 365 nm (*λ* excitation) and 445 nm (*λ* emission) and expressed in *μ*U/ngDNA using a standard curve of 4-methylumbelliferone from 50 nM to 10 *μ*M in 280 mM Tris-HCl Buffer pH 9.0.

#### 2.8.2. Calcium Mineralized Deposits Assay

Each well containing differentiated PA42-C4 cells on PCL film was incubated with 2 mL of 50 mM NaEDTA at room temperature, overnight, in order to solubilize all the calcium mineralized deposits. Then, the solution was transferred into a cuvette and the fluorescence measured with a spectrofluorometer LS55 (PerkinElmer) at 494 nm (*λ* excitation) and 517 nm (*λ* emission) and expressed in *μ*g/ng DNA, using a standard curve of hydroxyapatite from 25 ng/mL to 500 mg/mL, solubilized in 50 mM NaEDTA.

### 2.9. Statistical Analysis

For proliferation analysis, statistical processing was performed during the “log phase” of the growth curves using (a) the linearity test by Student's *t*-test and the *R* ^2^ coefficient of determination for each regression and (b) the parallelism test by Student's *t*-test to compare growth curves of PA42-C4 grown on PCL film with the growth curve of PA42-C4 grown on PS. For ALP and mineralized calcium deposits assays the experiments were carried out in quadruplicate and each experiment repeated three times. All data were expressed as means ± SD. Statistical differences among mean values were analyzed using Student's *t*-test.

All the results obtained on the biomaterial were compared with those obtained by growing the cells on polystyrene (PS) tissue culture, used as positive control. Data on PCL film were not significantly lower than those obtained on PS which were considered positive.

## 3. Results

### 3.1. Characterization of PA42-C4 Finite Clonal Line

The plastic-adherent cell population derived from human adipose tissue, called PA42, was cloned at passage 3. Thirteen clones were obtained and, among them, clone PA42-C4 was randomly selected for subsequent characterization.

**Figure 1:**
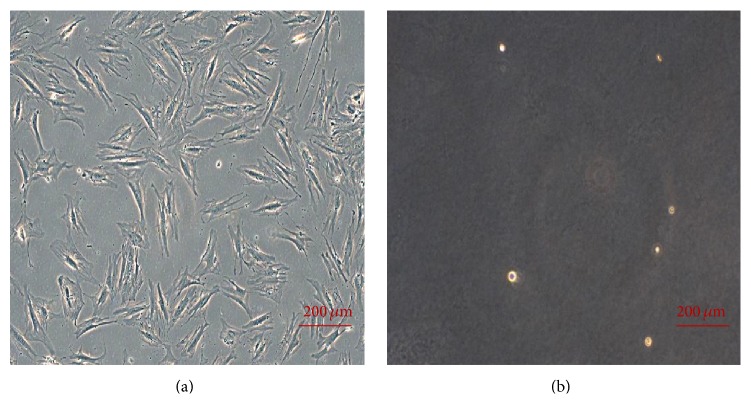
Representative images of the PA42-C4 clonal cell line grown on PS (a) and on soft agar (b). Phase contrast microscopy. Objective 10x.

The PA42-C4 cell line showed an elongated and fusiform, fibroblast-like shape, even if a greater number of cellular processes were present compared to the fibroblasts (Figure 1(a)). The PA42-C4 line did not show growth in soft agar after 4 weeks in culture, demonstrating no malignant transformation in cells (Figure 1(b)).

**Figure 2:**
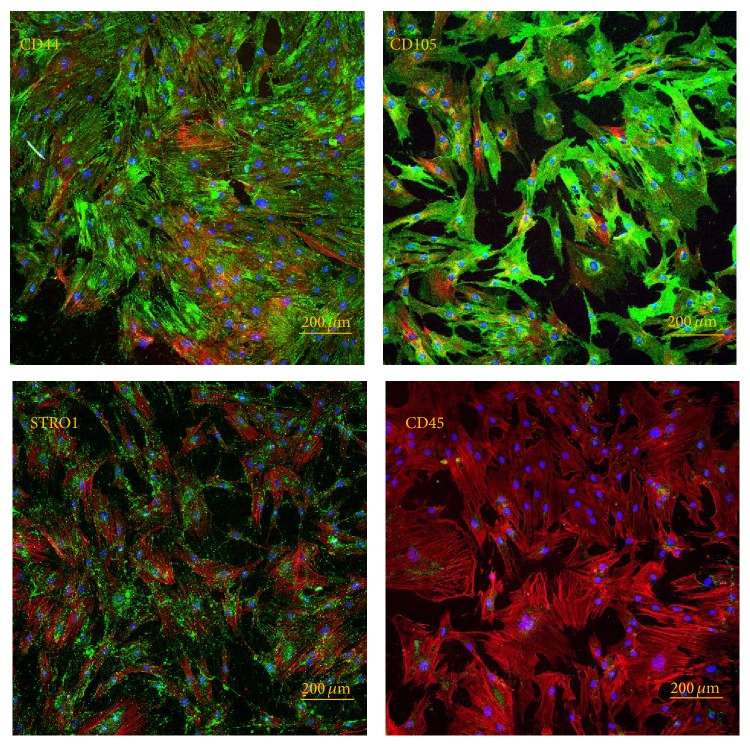
Microscopic observations in LSCM of the main stemness markers on hADSCs. Images show the positive immunofluorescence staining for CD44, CD105, and STRO1 (conventional green color) and negative immunofluorescence staining for CD45. Nuclei counterstained with propidium iodide (conventional blue color) and cytoskeleton with phalloidin-Alexa Fluor 635 (conventional red color). Objective 10x.

Immunofluorescent staining of PA42-C4, observed in LSCM, has allowed the expression of specific stemness markers on PA42-C4 surface, such as CD44, CD105, STRO1, and CD45, to be analyzed. The results obtained highlight the mesenchymal origin of the cell line, since CD44, CD105, and STRO1 were intensely stained, in contrast to the hematopoietic lineage marker CD45 which is negative (Figure 2).

The same positivity of the aforesaid markers was obtained with the heterogeneous population of PA42, before cell cloning (data not shown).

Afterwards, in order to verify their multipotency, both of the cell lines, PA42 and PA42-C4, were differentiated into adipogenic and osteogenic phenotypes.

**Figure 3:**
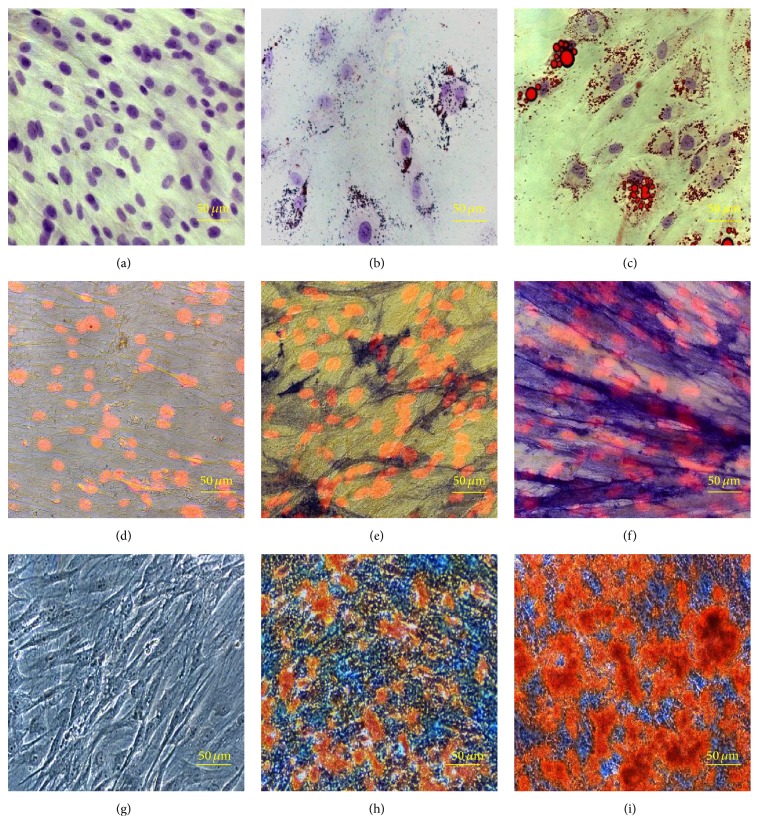
Multipotency evaluation of the PA42-C4 cell line. (a, b, c) Representative images of Oil Red O staining at time 0 and after 10 and 20 days of adipogenic induction; the intracellular lipids are in red and nuclei counterstained with Mayer's acid hemalum are in blue. Images acquired in brightfield microscopy. Objective 40x. (d, e, f) Representative images of Fast Blue BB staining at time 0 and after 10 and 20 days of osteogenic induction; ALP+ cells are in blue and nuclei counterstained with propidium iodide are in fluorescent red. Images acquired in brightfield microscopy for ALP+ cells and in epifluorescence for nuclei. Objective 40x. (g, h, i) Representative images of Alizarin Red S staining at time 0 and after 20 and 30 days of osteogenic induction; mineralized calcium deposits are in red. Images acquired in phase contrast microscopy. Objective 40x.

PA42-C4 induction with AM for 10 days resulted in the presence of small lipid droplets within some cells distributed around the cellular nucleus, indicating the beginning of the adipogenic differentiation. After 20 days, induction resulted in an expanded cell morphology and the accumulation of multiple intracellular lipid-filled droplets in a significant fraction of the cells (Figures 3(a), 3(b), and 3(c)).

Osteogenic induction of the PA42-C4 line was assessed with OM up to 20 days and observed during the entire period of study, monitoring the ALP expression and the production of mineralized calcium deposits.

Specifically, after 10 days, some cells began to express ALP, although with slight or medium intensity, surrounded by other negative cells. Finally, 20 days after the beginning of osteogenic induction, almost all of the PA42-C4 cells were strongly positive for ALP, with only a few, negative, scattered cells (Figures 3(d), 3(e), and 3(f)). Control cells grown in GM for the same time did not show any ALP+ cells.

Osteoblastic activity was evidenced by the production of an osteoid matrix by PA42-C4 cell line cultured in OM, obtaining an abundant deposition of a mineralized matrix after 20 and 30 days from induction, in a time-dependent manner. The area of matrix formation coincided with that of Alizarin Red S staining. In contrast, control cells grown in GM for the same time did not show any production of calcium deposits (Figures 3(g), 3(h), and 3(i)). Phase contrast microscopy observations of PA42-C4 cells stained with Alizarin Red S showed cell death and degeneration, observed near large mineralized deposits 35 days after osteoinduction.

The same behavior, using AM or OM, was confirmed in the heterogeneous population of PA42, before cell cloning (data not shown).

### 3.2. Preparation of PCL Film

PCL samples for cell culture experiments were prepared by solvent casting, a technique allowing the obtainment of polymeric films with limited thickness. This technique involves the dissolution of the polymer into a suitable solvent, the casting of the solution into a predefined mold, and the controlled evaporation of the solvent leaving behind a polymeric film. Homogeneous PCL samples were successfully prepared by optimizing the processing parameters, such as the polymer concentration and the volume of the cast solution. The use of an enclosed chamber saturated with solvent atmosphere during the drying process allowed slowing down the solidification process and thus the obtainment of a more uniform film surface morphology and thickness.

**Figure 4:**
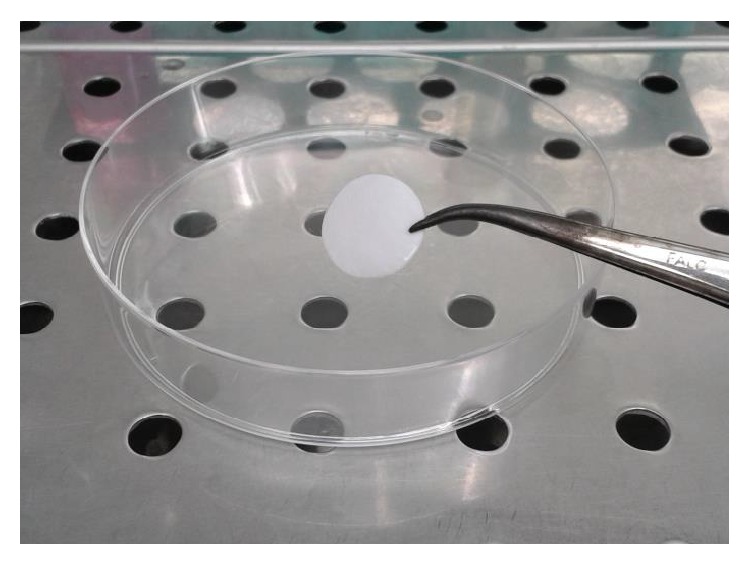
Representative photo of a disk of PCL film used in all of the experiments.

Specifically, the PCL film utilized for the study has a thickness of 70.35 ± 13.41 *μ*m (mean ± SD) (Figure 4).

### 3.3. Analysis of Cell Adhesion and Cytoskeletal Organization on PCL Film

**Figure 5:**
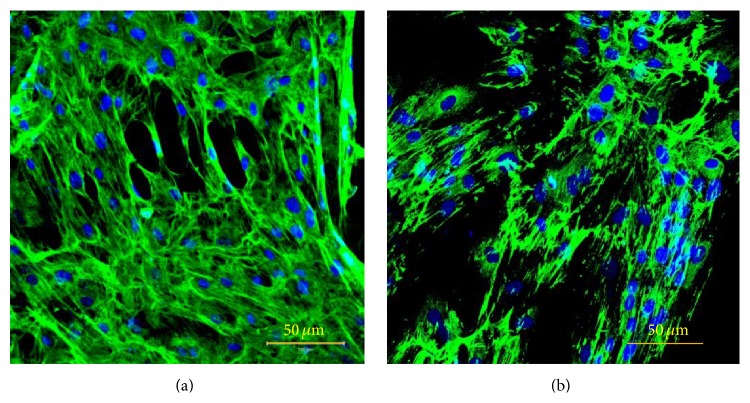
Microscopic observation in LSCM of fibronectin (Alexa Fluor 488, conventional green color) of PA42-C4 grown on PCL film (a) and on PS (b). Nuclei counterstained with propidium iodide (conventional blue color). Objective 20x.

Attachment of cells is one of the most important aspects of a scaffold. Microscopic examination in LSCM of fibronectin, the main cell adhesion protein, of PA42-C4 grown on PCL film for 7 days has permitted the verification of good adhesion of cells with intense staining localized over the entire cell surface, showing no qualitative differences with respect to PA42-C4 cells grown on PS, as control (Figure 5).

**Figure 6:**
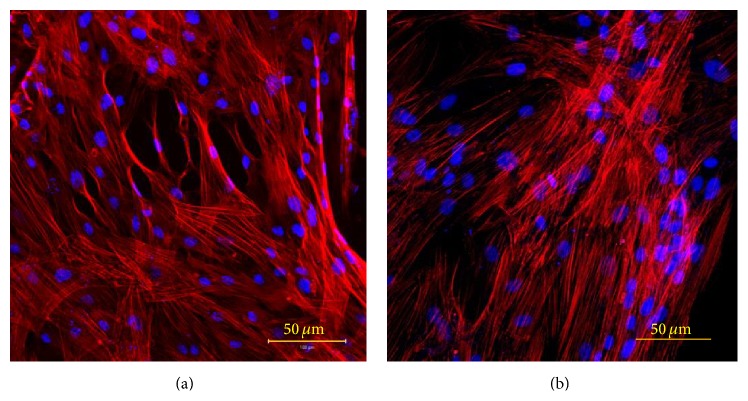
Microscopic observation in LSCM of cytoskeleton fiber (Alexa Fluor 635, conventional red color) of PA42-C4 cells grown on PCL film (a) and on PS (b). Nuclei counterstained with propidium iodide (conventional blue color). Objective 20x.

Cell morphology and cytoskeletal organization analysis, determined by F-actin labeling with phalloidin-Alexa Fluor 635, has demonstrated the presence of stress fibers in PA42-C4 grown for 7 days on PS and on a disk of PCL film. Cells appeared spindle- or rectangular-shaped with multiple cellular extensions and exhibited bundles of longitudinal F-actin filaments usually aligned along the major axis of the cell body. No significant differences in the cytoskeletal framework of PA42-C4 were found between the two supports (Figure 6).

### 3.4. Analysis of Cell Vitality and Proliferation on PCL Film

**Figure 7:**
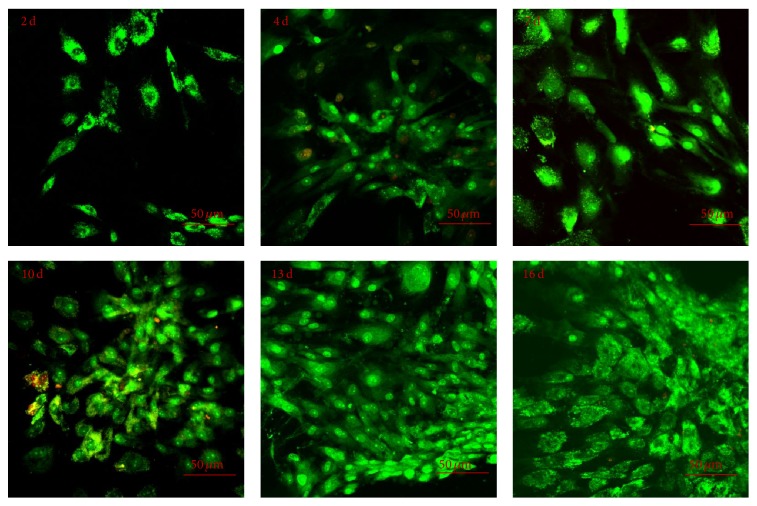
Representative images of PA42-C4 grown on PCL film at different experimental points (2–16 days) and stained with Acridine Orange. The nuclei and mitochondria appear green, while if cells are damaged or suffering, lysosomes should appear red-orange fluorescence colored. Objective 20x.

In order to assess the cytocompatibility and viability of cells on PCL film, the PA42-C4 line was stained with Acridine Orange and observed by fluorescence microscopy at different experimental points. The presence of red-orange emitted fluorescence attributed to lysosomes typically present in suffering or damaged cells was verified in less than 1% of the total cells, confirming the suitability of PCL film (Figure 7).

Subsequently, to confirm cell viability and proliferation on biomaterial, cells attached to film were quantified by the UV absorbance from the MTT assay, evaluating the mitochondrial activity, which proportionally increases with cell proliferation.

**Figure 8:**
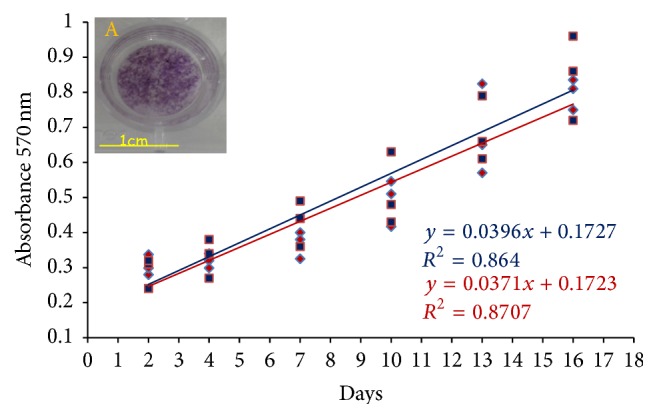
Analysis of the proliferation of PA42-C4 grown on PCL film (red) and on PS (blue), carried out at specific experimental points (2–16 days), using the MTT assay. The relative absorbance values were measured at 570 nm. The statistical analysis was performed by linearity test and parallelism test between the two regression lines. Image A shows a representative disk of PCL with violet crystals of formazan produced from MTT conversion by PA42-C4.

This assay showed a consistent increase in absorbance during all of the experimental period, confirming that good proliferation of the PA42-C4 on PCL film was achieved (linear regression *R* ^2^ = 0.87). These data are comparable and not significantly different compared to those obtained with the PA42-C4 grown on the substrate of control, PS (Figure 8).

### 3.5. Alkaline Phosphatase (ALP) Activity of PA42-C4 on PCL Film during Osteogenic Induction

Spectrofluorometric assay was performed for PA42-C4 grown on PCL film cultivated in osteogenic medium, in order to assess ALP activity at specific experimental times, from 0 to 35 days of osteoinduction.

Results have shown a significant increase of the ALP activity of PA42-C4 induced on PCL film from 14 days of induction, reaching a maximum value up to 21 days, correspondent to 41,53 *μ*U/*μ*g DNA with an increase of 7355% with respect to time 0. This increase substantially remained constant at 28 days and then decreased after 35 days of induction.

**Figure 9:**
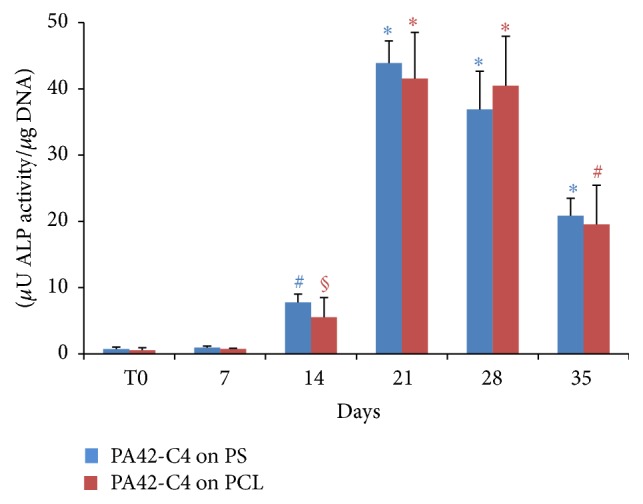
Analysis of ALP activity in PA42-C4 induced on PCL film (red) and on PS (blue) from 0 to 35 days. The values are the mean ± SD of three independent experiments. Significance was obtained by Student's *t*-test, comparing the value of ALP activity to the different time points with respect to time 0, for cells induced on PCL film or on PS. ^*∗*^ *p* < 0.001 versus respective time 0; ^#^ *p* < 0.005 versus respective time 0; ^§^ *p* < 0.05 versus respective time 0.

A similar pattern, not significantly different with respect to PCL film, was obtained in PA42-C4 induced on PS, as control, during the entire study period (Figure 9).

**Figure 10:**
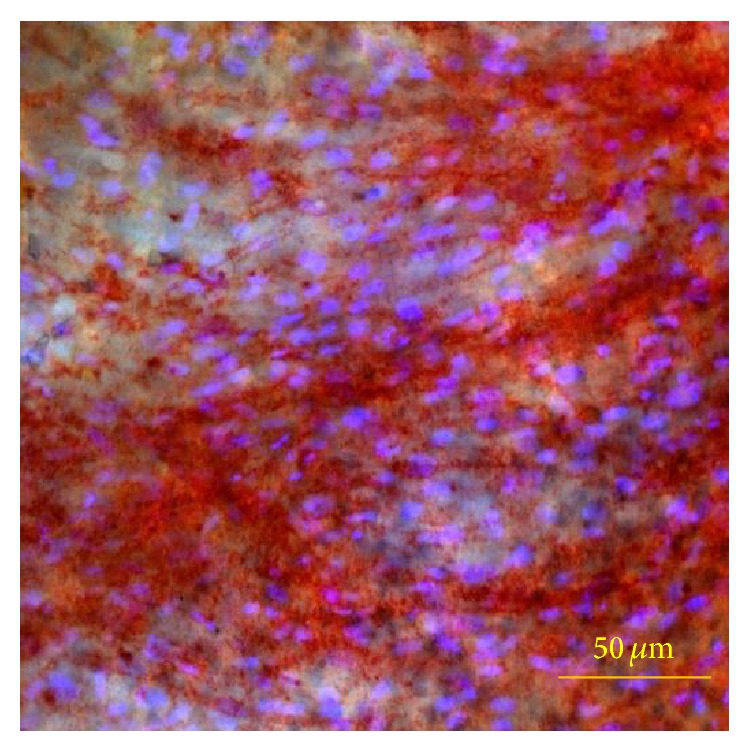
Representative observations of the PA42-C4 line on PCL film induced in osteogenic medium up to 35 days. ALP+ cells are stained with Fast Red Violet LB (red color) and nuclei with DAPI (fluorescent blue color). Image acquired in brightfield microscopy for ALP+ cells and in epifluorescence for nuclei. Objective 20x.

The ALP activity was observed in brightfield microscopic, using a high lamp voltage in order to make the ALP+ cells visible through the thickness of the PCL film. The ALP+ cells are evident thanks to the Fast Red Violet LB staining, as previously described in Materials and Methods (Figure 10).

### 3.6. Mineralization Analysis of PA42-C4 on PCL Film during Osteogenic Induction

The formation of mineralized extracellular matrix was assessed by spectrofluorometric assay, quantifying the production of calcium deposits at different experimental points, from 0 to 35 days of osteoinduction. The quantity of calcium deposits obtained by the seeded cells and induced on PCL film was compared to that obtained for PA42 differentiated on substrate control, PS.

The obtained results have shown a significant increase in the production of calcium nodules by PA42-C4 induced on PCL film starting at 14 days, reaching the maximum value at 35 days, corresponding to 111,53 ng/*μ*g DNA, with an increase of 13337% with respect to time 0.

**Figure 11:**
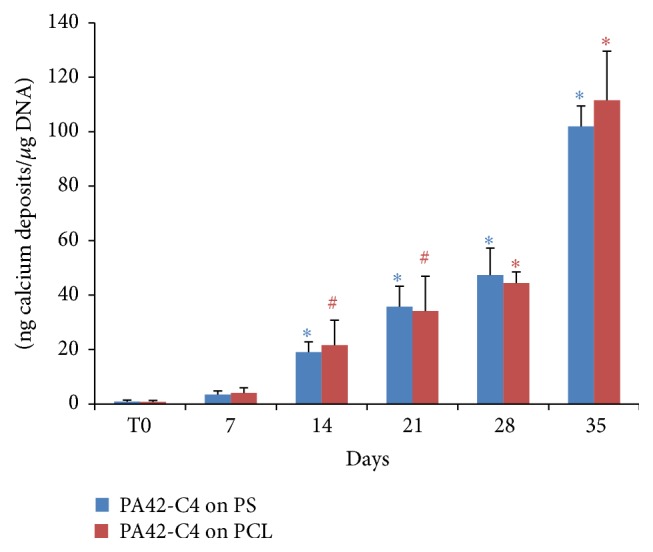
Analysis of mineralized calcium deposits in PA42-C4 induced on PCL film (red) and on PS (blue) from 0 to 35 days. The values are the mean ± SD of three independent experiments. The significance was evaluated by Student's *t*-test comparing the value of calcium deposits at different experimental points with respect to time 0 for cells induced on PCL film or on PS. ^*∗*^ *p* < 0.001 versus the respective time 0; ^#^ *p* < 0.005 versus the respective time 0.

A similar pattern, not significantly different with respect to PCL film, was obtained in PA42-C4 induced on PS, as control, during the entire study period (Figure 11).

**Figure 12:**
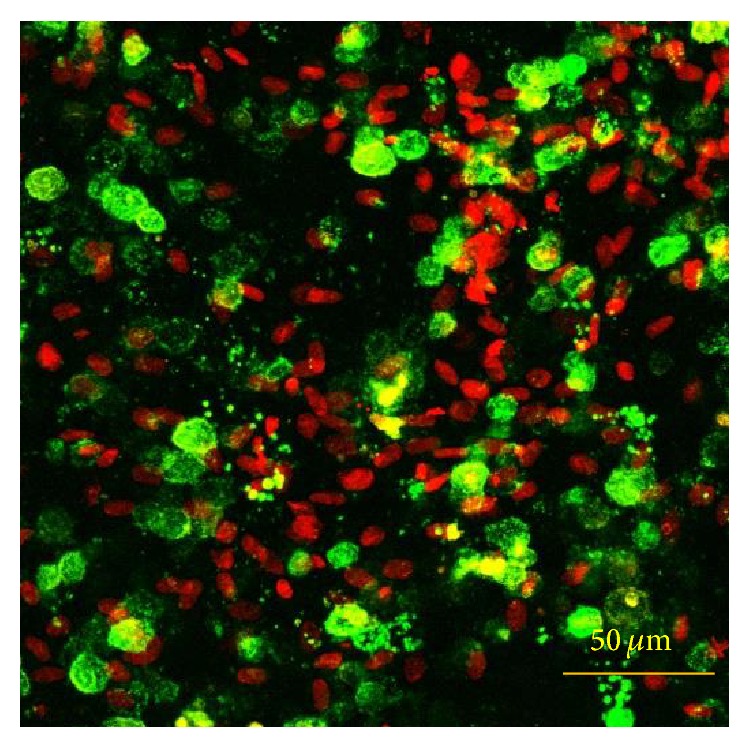
Representative observations acquired in LSCM of the PA42-C4 line on PCL film induced in osteogenic medium up to 35 days. Calcium deposits are stained with calcein (conventional green color) and nuclei with propidium iodide (conventional red color). 3D projection of Z-Stacks of 23 optical sections with an interval of 3 *μ*m from each other. Objective 20x.

The production of calcium deposits was observed by microscopic observation acquired in LSCM; in fact, calcium nodules are evident thanks to the addition of fluorophore calcein to the osteogenic medium during the entire experimental period (Figure 12).

## 4. Discussion

Multipotent cells derived from adipose tissue represent an attractive tool in regenerative medicine. The higher number of hAMSCs yielded from lipoaspirates, able to differentiate into mesodermal lineages (osteogenic, adipogenic, chondrogenic, and myogenic phenotypes) as well as the interesting immunomodulating properties, implicit in MSCs, suggests that they can be considered very promising for future application in cell-based therapy.

In this study, we have isolated and characterized a single-cell-derived clone of hAMSCs, carried out for investigating the potential of a homogeneous population of MSCs in order to obtain more coherent results with respect to the use of a heterogeneous subset of cells obtained from adipose tissue, as discussed by Guilak et al. [41]. Our results have shown that the clone PA42-C4 has maintained the multipotentiality of the heterogeneous population PA42, demonstrating its own capacity to differentiate into the adipogenic and osteogenic phenotypes, as assessed by the cytochemical staining performed on cells, properly induced with specific differentiating media. Moreover immunofluorescence analysis has permitted the verification of the presence of the principal markers largely recognized to be expressed on the surface of MSCs, specifically CD44, CD105, and STRO1, confirming the validity of our clone for further experiments.

Subsequently, we have analyzed the* in vitro* behavior of the clonal human cell line, PA42-C4, in combination with PCL film, since PCL has been widely investigated for tissue regeneration applications. Nevertheless, there are no studies in literature which take into consideration the above cellular type and 2D PCL film. So, we have demonstrated the potential of this cell-biomaterial model for future application in bone tissue engineering.

PCL has good biocompatibility, inexpensive production routes, tuneable biodegradation kinetics and mechanical properties, and good blend-compatibility. In addition, by way of its superior rheological properties and ease of shaping, PCL has incredible processing versatility being suitable for a wide range of techniques investigated for tissue engineering scaffold fabrication [33]. Indeed, a number of processing techniques have been applied to process PCL into scaffolds, such as particulate leaching, phase separation, textiles, and additive manufacturing techniques. This allows designing and manufacturing PCL scaffold with tailored structural features at the macro-, micro-, and nanoscale levels, such as foam [42], microfiber constructs with a predefined network of pores [43], and nanofibrous assemblies with high surface area [44].

The ability of cells to recognize and interact with the selected support represents the first essential step, without which processes such as cell proliferation, migration, and differentiation would not be possible. It is generally accepted that well-formed actin stress fibers and adhesion would infer stable attachment and cell survival on material surfaces [45]. For that purpose, in our study, we have verified the expression of the adhesion protein fibronectin and the organization and morphology of the cytoskeleton of PA42-C4 cells grown on PCL film, demonstrating the achievement of good cell adhesion to the biomaterial. Our results have not shown any significant differences compared to those observed in cells grown on PS, used as control.

Afterwards, we assessed cell viability/proliferation on biofilm in order to confirm not only the cytocompatibility of the biomaterial, but also that PA42-C4 cells are able to proliferate on PCL film. We have found that the proliferation process increases in a time-dependent manner, with no significant differences compared to cells grown on PS, as control, bringing evidence that PCL film does not affect PA42-C4 proliferation capacity.

Finally, since bone regeneration represents our target, to understand the osteogenic differentiation of PA42-C4 on PCL film and demonstrate the potential of our proposed model, we have evaluated ALP activity and calcium deposition; these parameters are widely used as markers for early and late differentiation of osteoblast-like cells, respectively. Results obtained have indicated that PCL film has supported phenotypic differentiation of hAMSCs by triggering the enzyme's activity and the production of a mineralized extracellular matrix, confirming the suitability of the aforementioned biomaterial to ensure the osteoblastic differentiation process.

Overall, although the results obtained on PCL film are not significantly different from those obtained by growing and inducing cells on PS, used as control, the encouraging results of this study in terms of cell adhesion, proliferation, and differentiation of hAMSCs on PCL film make the hAMSCs/PCL film a suitable model for further investigation, opening new possibilities for the development of engineered constructs with tailored structure, optimized for the regeneration and 3D* in vitro* models of bone tissue.

Current literature reports the development of numerous techniques of surface modification to increase cell adhesion on synthetic polymers by modifying polymer surface morphology (roughness) and/or hydrophilicity and by immobilizing specific ECM derived peptides. In fact, it is well demonstrated that these variations could improve the interaction between cell and substrate, high cell viability, and enhance bone formation capability [36, 38, 45–47]. Moreover, over the last decade, since polymers lack osteoconductivity, considerable attention has been directed toward their combination with natural or synthetic bioceramics, such as hydroxyapatite or calcium phosphate. This strategy has permitted the creation of many composite scaffolds that imitate the natural structure of bone with superior osteoconductive property, stimulating MSCs to foster a favorable osteogenic microenvironment [31, 40, 48, 49].

Keeping all of this in mind, it seems, therefore, reasonable to carry out further studies to improve the properties of our proposed model, hAMSCs/PCL film, for future application in bone tissue regeneration.

## 5. Conclusion

This study has demonstrated that hAMSCs can be grown and induced to differentiate in osteoblastic cells on PCL film prepared by solvent casting, suggesting and promoting research of new strategies to enhance and maximize the cellular reply and differentiation on the substrate for future use in bone tissue engineering applications. However, it is still evident that in order to achieve more conclusive results further* in vivo* studies are needed to optimize the biological mechanism for osseous integration and regeneration using the proposed cell-biomaterial model.
